# THE INFLUENCE OF MEDIA PORTRAYALS ON AGEISM: A CASE STUDY OF THE GOLDEN BACHELOR

**DOI:** 10.1093/geroni/igae098.0013

**Published:** 2024-12-31

**Authors:** Sarah Fraser, Alixe Menard, Alison Chasteen, Mateja Van Gameren, Chris Belanger

**Affiliations:** University of Ottawa, Ottawa, Ontario, Canada; University of Ottawa, Ottawa, Ontario, Canada; University of Toronto, Toronto, Ontario, Canada; Lisgar Collegiate Institute, Ottawa, Ontario, Canada; University of Ottawa, Ottawa, Ontario, Canada

## Abstract

Background: Age-based discrimination or ageism is often propagated through media platforms (TV, news, social media). Focusing on the reality television show “The Golden Bachelor,” which features older adult contestants seeking romance, this study explored ageism spread via social media posts related to the program. Methods: Using social astronomy software we scraped Reddit’s English-language corpus for posts containing “golden bachelor” and extracted a sample of 1000 posts shared between August and September 2023. Qualitative content analysis of these posts revealed four forms of ageism (1) personal, (2) explicit, (3) implicit, and (4) benevolent. Results: Personal ageism emerged through comments devaluing older contestants’ appearance: “Hey at 75 you can go on The Golden Bachelor; wouldn’t that be fun. Wrinkles upon wrinkles…” Explicit ageism was evident in derogatory remarks targeting contestants’ age and limited physical capabilities: “Can the Golden Bachelor still get it up?”. Implicit ageism was discernible in subtle reinforcement of age-related stereotypes: “The guy who’s going to be the Golden Bachelor is 71 and he looks much younger than I remember 71-year-olds looking. He seems like a pretty normal guy.” and benevolent ageism manifested through the infantilization of older contestants: “cute old people love”. Discussion and Implications: While “The Golden Bachelor” may help some understand the capabilities of older adults and counter the misperception of them as asexual, existing ageist stereotypes emerge and are propagated on social media platforms. These findings underscore the pervasive nature of ageism in social media and highlight the importance of addressing age-related biases in media representation.

